# Convalescents

**Published:** 1907-04-27

**Authors:** 


### CORRESPONDENCE.

### Convalescents.

The Rev. Herbert E. Gunson, chaplain of the Middlesex Hospital, writes:—In your issue of the 6th inst. I have seen your kind notice of the valedictory service held in the chapel of this hospital for patients about to proceed to our convalescent home at Clacton-on-Sea. Will you allow me to correct a slight mistake in your paragraph? The service is not held once a fortnight, but every week, and although intended primarily for the patients proceeding to our Clacton Home, we welcome all other patients, and, indeed, all who are able to attend. The service has been held regularly without break for several years, and never exceeds half an hour in length.

